# Reducing length of stay and satisfying learner needs

**DOI:** 10.1007/s40037-016-0276-2

**Published:** 2016-05-31

**Authors:** Lisa Shepherd, Saad Chahine, Michelle Klingel, Elaine Zibrowski, Allison Meiwald, Lorelei Lingard

**Affiliations:** Centre for Education Research and Innovation, Schulich School of Medicine & Dentistry, Western University, London, Ontario Canada; Division of Emergency Medicine, Department of Medicine, Western University, London, Ontario Canada

**Keywords:** Undergraduate medical education, Emergency department wait times, Self-efficacy

## Abstract

A complicated relationship exists between emergency department (ED) learner needs and patient flow with solutions to one issue often negatively affecting the other. Teaching shifts that allow clinical teachers and learners to interact without the pressure of patient care may offer a mutually beneficial solution. This study investigated the relationship between teaching shifts on ED length of stay, student self-efficacy and knowledge application.

In 2012–2013, a prospective, cohort study was undertaken in a large Canadian acute-care teaching centre. All 132 clinical clerks completing their mandatory two-week emergency medicine rotation participated in three teaching shifts supervised by one faculty member without patient care responsibilities. The curriculum emphasized advanced clinical skills and included low fidelity simulation exercises, a suturing lab, image interpretation modules and discussion about psychosocial issues in emergency medicine. The clerks then completed seven clinical shifts in the traditional manner caring for patients under the supervision of an ED attending physician. Length of stay was compared during and one week following teaching shifts. A self-efficacy questionnaire was validated through exploratory factor analysis. Pre/post knowledge application was assessed using a paper-based clinical case activity.

Across 40.998 patient visits, median length of stay was shortened overall by 5 minutes (95 % CI:1.2, 8.8) when clerks were involved in their teaching shifts. In the first academic block, median length of stay was reduced by 20 minutes per patient (95 % CI:12.7, 27.3). Self-efficacy showed significant improvement post teaching shifts (*p* < 0.001) with large effect sizes (d > 1.25) on dimensions of knowledge base, suturing, trauma and team efficacy. Students’ knowledge application scores improved from pre to post (*p* < 0.01), with notable gains in the generation of differential diagnoses.

Teaching shifts are an effective educational intervention that has a positive relation to ED patient flow while successfully attending to learner needs. Teaching shifts for the most naïve clerks in the first academic block appear to maximally benefit length of stay. Students demonstrated improved self-efficacy and knowledge application after their teaching shifts.

## What this paper adds

A complicated relationship exists between emergency department (ED) learner needs and patient flow with solutions to one issue often creating negative consequences for the other. This paper describes one emergency department’s educational intervention – teaching shifts for clinical clerks – where these students receive small-group instruction by emergency medicine physicians over three days in a setting without patient care responsibilities. The relationship of these teaching shifts to ED length of stay, student self-efficacy and knowledge application offers support to the concept that educational interventions in the ED can benefit both learners and patient flow.

## Introduction

One of the ubiquitous challenges of clinical training is to maximize the benefits of direct patient contact for learners while minimizing the drawbacks of learner involvement for the healthcare system. Learners can slow down the process of care provision with important consequences for health economics [1]. In an era of strained healthcare resources, effective educational strategies to address this problem are urgently needed.

For the emergency department (ED) physician working in an academic healthcare centre, this challenge has become paramount. Over the past 20 years the constant tension between the needs of the learner and patient flow has been further compounded by ED overcrowding, which has focused a great deal of institutional and government attention on the issue of patient flow, arguably at the expense of their educational mandate. In a recent Canadian study, Webster et al. [2]. found that government policies aimed at reducing ED wait times left medical students and junior doctors with the impression that efficiency is more important than education.

A complicated relationship exists between the needs of ED learners and patient flow. While studies report that trainees prolong the length of stay of ED patients [3–5], there is little known about the reciprocal effect of overcrowded EDs on the educational environment. What is known suggests differing perspectives from teachers and learners. Anecdotally, many teachers believe that poor patient flow has been detrimental to learning in the ED. This is supported by a study in which teachers perceived ED overcrowding to limit bedside teaching opportunities [6], although lack of research on ED teaching prior to overcrowding makes comparative claims impossible [7]. Concern has been expressed that in crowded EDs, learners may lower their standards for clinical care, patient privacy and professionalism [8]. However, residents perceived minimal impact on educational value during times of overcrowding, although they saw fewer patients and performed fewer procedures [9]. Analyses of learner evaluations of ED attending physicians suggested that overcrowding did not change learner satisfaction with teaching [10]. Additionally, there are suggestions that poor patient flow may present affordances as well as threats; for instance, Shayne et al. [11] argued that crowded EDs may offer learners in-depth opportunities to focus on multitasking, communication skills, professionalism and system-based competencies.

Because of the complicated relationship between ED learning and patient flow, solutions directed at one of these issues in isolation can have unintended negative consequences on the other. For example, ED Fast Track areas for low acuity patients [12, 13], which have been shown to positively influence patient flow, often restrict or entirely exclude junior learners, thereby limiting their exposure to the assessment and procedure opportunities associated with this subgroup of patients.

Given the importance of the ED as a learning environment, we contend that it is imperative to develop effective educational solutions that are mindful of the issue of patient flow. ED teaching shifts may represent such a solution. An ED teaching shift is a shift scheduled for the sole purpose of learning, where students and teacher have no patient care responsibilities. The literature describes great variety in the composition and delivery of these teaching shifts [14–16]based upon the needs of a particular programme. However, we have found no study to date examining how such a strategy affects ED throughput or the educational experience.

The aim of this study is to examine how one educational intervention, ED teaching shifts, relates to length of stay, learner self-efficacy and knowledge application. Our study addressed the following three questions:

1. How do ED teaching shifts relate to emergency department length of stay?

Emergency department length of stay is often used as a metric of efficient ED patient flow and is defined as the time in minutes from arrival at triage or registration until discharge from the ED. It is dependent on timeliness of multiple interrelated elements such as offloading of ambulance patients, triage, nurse and physician assessment, investigations and availability of in-patient hospital beds.

2. How do ED teaching shifts relate to the self-efficacy of clinical clerks?

Self-efficacy is defined as a personal judgment about how well someone can organize and execute a course of action required to deal with a prospective situation [17]. Important features of the definition are context specificity and prospective action, which distinguish self-efficacy from other concepts such as self-esteem, self-assessment, self-concept and self-confidence. Increasing levels of perceived self-efficacy give rise to progressively higher performance accomplishment [18]. Self-efficacy is a meaningful measure for a study of ED student learning, as it can affect rate of skill acquisition and performance mastery, which in turn can boost self-efficacy in a mutually enhancing process [17].

3. How do ED teaching shifts relate to knowledge application by clinical clerks?

Not yet at the point of sophisticated clinical reasoning, clinical clerks are challenged to apply the knowledge gained in their limited clinical experience and preclinical years to a variety of undifferentiated emergency patient presentations. Using a well-defined rubric, assessment of a post encounter exercise has shown validity in assessing medical content as well as logic and thought processes [19].

## Method

### Setting

The study setting was a two-site, 1050 bed academic health centre in London, Ontario, Canada with a combined adult ED annual census of 114,000 patient visits. The emergency medicine medical student clerkship is a two-week mandatory rotation in the third year of a four-year programme. Teaching shifts, two 8‑hour and one 4‑hour, were delivered during the first three days of their rotation supervised by a different faculty member each day who did not have patient care responsibilities. Ten faculty members with an interest in teaching and between 1 and 25 years of teaching experience volunteered to give these sessions throughout the year. They were compensated using the standard departmental teaching rate.

The curriculum, shown in Tab. 1, was informed by a literature review and survey of other clerkship programmes locally and emergency medicine clerkship programmes nationally. It emphasized advanced clinical skills, with students seeing previously assessed ED patients who were awaiting admission or further testing. Student presentations of these patients were reviewed to teach both basic differential diagnosis, including significant emergent worst-case scenarios, and ED-appropriate investigation and treatment plans. The curriculum also contained two low-fidelity simulation exercises dealing with basic cardiac and trauma resuscitation, a suturing lab, image interpretation modules and small-group discussions on selected psychosocial issues in emergency medicine. Following the teaching shifts, students completed seven 8‑hour clinical shifts in the traditional manner, caring for patients under the supervision of an ED attending physician. This involved students obtaining a history and performing a physical exam on assigned patients in the ED followed by a case presentation to either their attending physician or a senior emergency medicine resident. An investigation and treatment plan are decided upon together and the patient is followed through until disposition.

**Tab. 1:** Teaching shift curriculum

Day	Activity	Time (in h)
1	Orientation to site 1 ED and emergency medicine rotation	1
(at site 1)	^a^Advanced clinical skills – abdominal pain	2
	Lunch – psychosocial learning topics explained and assigned	1
	^a^Advanced clinical skills – abdominal pain	1
	Suturing/wound care skills session	2
2	Orientation to site 2 ED	0.5
(at site 2)	Advanced clinical skills – musculoskeletal	1.5
	The first 10 minutes of trauma – simulation	1
	Psychosocial topic discussion	1
3		
(at site 2)	^a^Advanced clinical skills – chest pain and shortness of breath	2
	The first 10 minutes of cardiac emergency – simulation	2
	Lunch – psychosocial topic discussion	1
	^a^Advanced clinical skills – chest pain and shortness of breath	2
	Wrap up – evaluations	1

^a^During the first hour of these morning advanced clinical skills sessions, one half of the group would see previously assessed ED patients for a brief history and focused physical exam while the other half participated in image interpretation independent learning modules. The groups would then come together for the case presentation and discussion in the second hour. In the afternoon, the process would be repeated with the groups reversed

After obtaining Western University Research Ethics Board formal exemption from ethical review, a prospective cohort study was conducted during the 2012/2013 academic year. All statistical analysis was conducted using Stata IC 13.1 or SPSS 22.

### Measures

#### Length of stay

In 2004, the Ontario Ministry of Health and Long Term Care launched the Ontario wait time strategy. As part of this project, the Emergency Room National Ambulatory Care Reporting System (NARCS) Initiative (ERNI) was implemented in 2010, whereby hospitals are required to collect and report a number of ED metrics including length of stay. Between September 2012 and August 2013, length of stay times were collected from hospital ERNI data on Mondays, Tuesdays and Wednesdays when the students were out of the ED and taking part in their teaching shifts (teaching shift period). To minimize confounding variables such as staffing patterns, availability of ancillary services, seasonal variation, and implementation of department and hospital-based patient flow initiatives, the study compared data from these three teaching shift days with Mondays, Tuesdays and Wednesdays of the week immediately following when the same students were present in the ED, participating in patient care (ED period). The total number of ED visits was determined for both the teaching shift and the ED periods and median length of stay times were calculated for each.

#### Self-efficacy

To measure change in a learner’s belief in ability, we asked students to report their self-efficacy pre and post teaching shift. Students completed a 20-statement, self-efficacy questionnaire, in the first and last hour of the teaching shifts, rated on a 7-point Likert scale (1 = strongly disagree, 7 = strongly agree) reflecting specific knowledge and skills covered in the curriculum.

#### Knowledge application

An objective assessment of student knowledge application was administered pre and post intervention using a paper-based clinical case of a patient presenting with either undifferentiated chest or abdominal pain. On the first morning of the teaching shifts students were asked to record on paper pertinent features from the history and physical examination and to formulate a differential diagnosis and an ED-appropriate investigation and management plan. They were then asked to repeat this at the end of the third teaching shift with a different paper-based case, matched to the same presenting complaint (chest pain or abdominal pain). These pre and post case exercises were then independently scored by two blinded faculty using a modified, previously validated scoring rubric [20]. This 9‑point rating rubric was based on a holistic overview of the written information, ranging from 1 (clearly unacceptable) to 9 (clearly superior). In instances where the difference in scores was ≥ 2, the exercises were re-graded by the same faculty and, where necessary, discussed and adjusted to resolve the score difference to less than two.

### Analysis

#### Length of stay

Differences between medians with 95 % confidence intervals surrounding the differences were calculated using the Bonett-Price method [21]. The results were grouped into four blocks, corresponding to the quarterly rotations throughout the year to explore temporal changes with clerkship experience.

#### Self-efficacy

Descriptive statistic, reliability and factor analysis were first conducted as part of the validation processes. Based on the dimensionality of the instrument, scale scores were produced. Paired t‑tests with 95 % confidence intervals and Bonferroni corrections were used to evaluate the magnitude of change.

#### Knowledge application

To explore the change in conceptualization and thinking about the generation of a differential diagnosis list, we first conducted a paired t‑test followed by an examination of the qualitative change. The results and sample of the conceptual change are provided in the results.

## Results

During the 2012/2013 academic year, there were 24 groups of 6–7 clinical clerks arriving every two weeks for their emergency medicine rotation. The year was divided into four blocks with block 1 being the start of the clerkship and block 4 completing the year. There were no clinical clerks in the ED during the first and last week of the academic year. In each of December (block 2) and March (block 3), one of the two-week rotations was extended to three weeks to accommodate holidays. These two groups were excluded leaving 22 groups available for analysis. From September 2012 until August 2013, 132 clinical clerks participated in three teaching shifts.

### Length of stay

Over the one-year study period, 40,998 patient visits occurring on Mondays, Tuesdays and Wednesdays were analyzed to determine ED length of stay, with 20,538 of these visits taking place during the time the students were in their teaching shifts (Tab. 2). Overall, median length of stay was shorter by 5 minutes (241 vs. 246 minutes; 95 % CI: 1.2, 8.8) when students were involved in their teaching shifts. In block 1 (least experienced clerks), median length of stay was shorter by 20 minutes (95 % CI: 12.7, 27.3) in the teaching shift period. In block 2 and block 4, no statistically significant difference was found between times when clerks were in teaching shifts or in the department. In block 3, median length of stay was 7 minutes longer (95 % CI: 4.4, 9.6) when the clerks were in their teaching shifts.

**Tab. 2:** Emergency department length of stay (EDLOS)

	EDLOS			
Rotations	*n* ^a^	Median^b^	Diff^b^	95 % CI
*Block 1*				
Clerks in ED	5694	263		
Clerks in teaching shifts	5544	243	−20	−27.3, −12.7
*Block 2*				
Clerks in ED	4466	245		
Clerks in teaching shifts	4533	250	5	−4.4, 14.4
*Block 3*				
Clerks in ED	4606	233		
Clerks in teaching shifts	4749	240	7	4.4, 9.6
*Block 4*				
Clerks in ED	5694	237		
Clerks in teaching shifts	5712	235.5	−1.5	−9.5, 6.5
*Overall*				
Clerks in ED	20,460	246		
Clerks in teaching shifts	20,538	241	−5	−8.8, −1.2
Total	40,998			

*n* number; *diff* difference; *CI* confidence interval

^a^number of ED visits

^b^time measured in minutes

### Self-efficacy

There were 122 matched pre/post pairs available for analysis of self-efficacy. To develop dimension summaries, Kaiser-Meyer-Olkin (KMO) value pre/post (0.902/0.814) and Bartlett’s test of sphericity (1258.10/934.017) suggested that exploratory factor analysis (EFA) was appropriate given our sample size-to-item ratio. An EFA with maximum likelihood estimation with varimax rotation resulted in a four-factor solution representing dimensions of knowledge base, suturing, trauma and team. Using an approach developed by Osborne & Fitzpatrick [22], items where the squared difference of the pre/post factor loadings was greater than 0.02 were removed. Tab. 3 outlines the items in the survey and the factor structure as well as the items that were included and removed. Additionally Cronbach’s alpha for each of the three dimensions (Suturing 0.82/0.80, Team 0.71/0.70, Trauma 0.79/0.71) were high; however, knowledge base post reliabilities (0.71/0.50) was slightly lower. Averages were used to represent the level of perceived efficacy on each dimension and t‑tests confirmed a significant increase in self-efficacy from pre to post (Tab. 4).

**Tab. 3:** Factor analysis of self-efficacy statements

	Pre/Post survey			
*Included statement*	*K-base*	*Suturing*	*Teams*	*Trauma*
1. I know how to give a report to another member of the healthcare team who is about to take over the care of a patient I have looked after	0.55/0.65			
2. I am able to assess an acutely injured knee	0.54/0.41			
3. I know how to recognize and initiate treatment for hypovolaemic shock	0.50/0.45			
4. I am able to suture a simple skin laceration		0.76/0.73		
5. I am able to infiltrate a wound with local anaesthetic		0.69/0.72		
6. I know how to choose an appropriate local anaesthetic for suturing		0.61/0.57		
7. I know when a wound should NOT be sutured in the ED		0.58/0.71		
8. I am able to lead a team in a basic resuscitation situation			0.79/0.98	
9. I am able to communicate effectively as part of a resuscitation team			0.75/0.61	
10. I know how to call a ‘Code Blue’ in any London Hospital			0.44/0.45	
11. I am able to apply cervical spine precautions in a patient who may have a cervical spine fracture, both inside and outside of the hospital setting				0.82/0.82
12. I am able to implement the Canadian C‑Spine rules				0.59/0.50
13. I am able to direct and assist in log rolling a trauma patient				0.44/0.55
*Removed statement*	*K-base*	*Suturing*	*Teams*	*Trauma*
14. I am able to perform a primary survey in a trauma patient	0.76/0.51		–/0.34	
15. I am able to perform bag-valve mask ventilation	0.58/–			
16. I know how to calculate a patient’s Glasgow Coma Score	0.49/–			
17. I am able to generate an appropriate list of differential diagnoses in a patient with chest pain presenting to the ED	–/–			
18. I know how to make a plan for the investigation and treatment of a patient presenting to the ED with abdominal pain	0.43/–	0.48/–		
19. I am able to defibrillate a patient in cardiac arrest at the appropriate time for the appropriate rhythm	–/0.39		0.55/0.33	
20. I am able to demonstrate all basic life support skills in the appropriate sequence	0.53/–			–/0.50

Factor loadings <0.30 are suppressed

*K-base* knowledge base

**Tab. 4:** Pretest/posttest self-efficacy dimensions

	Pretest			Posttest			98.75 % CI for mean difference			
Outcome	M	SD		M	SD	*n*		t	df	Cohen’s D
Knowledge base	4.71	1.02		5.86	0.56	122	0.92,1.37	13.11**	121	1.19
Suturing	4.00	1.26		5.45	0.97	122	1.18,1.71	13.82**	121	1.25
Teams	2.67	1.11		5.55	0.85	122	2.59,3.16	25.50**	121	2.30
Trauma	3.31	1.33		5.54	0.83	122	1.93,2.56	18.04**	121	1.63

98.75 confidence interval calculated using Bonferroni adjusted alpha (0.05/4); ** *p* < 0.001

### Knowledge application

**Fig. 1:**
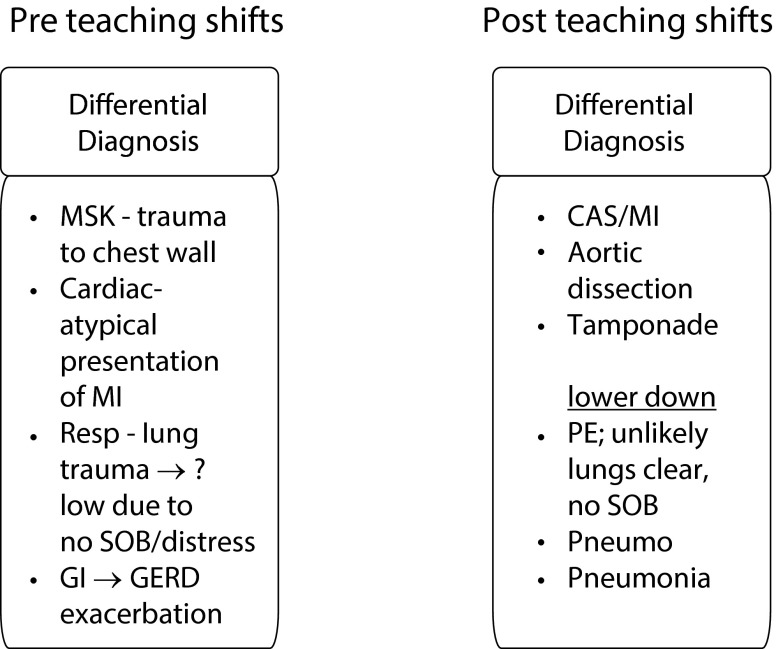
Pre/post teaching shifts differential diagnosis paper-based case contrasts: Student A.* (MSK* musculoskeletal, *MI* myocardial infarction, *resp* respiratory, *SOB* shortness of breath, *GI* gastrointestinal, *GERD* gastroesophageal reflux disease, *CAS[ACS]* acute coronary syndrome, *PE* pulmonary embolus, *pneumo* pneumothorax)

**Fig. 2:**
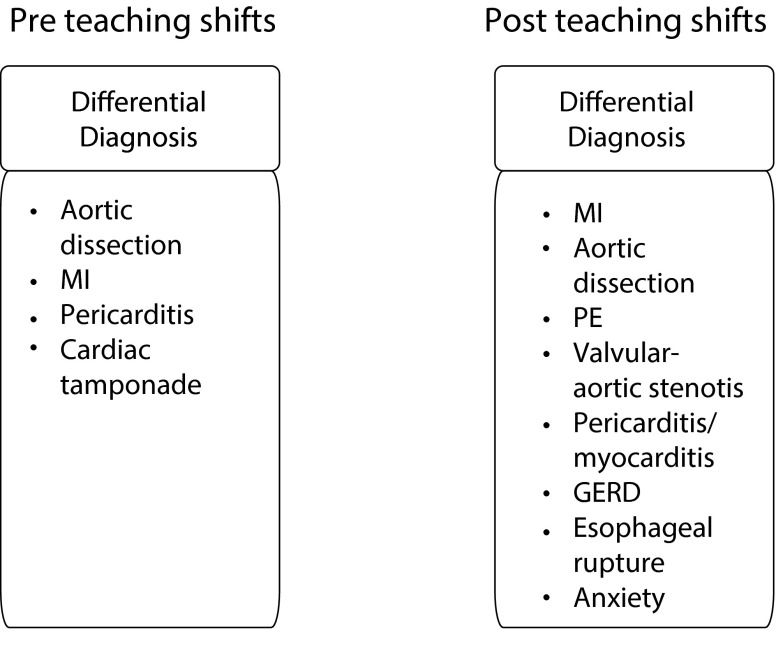
Pre/post teaching shifts differential diagnosis paper-based case contrasts: Student B*. (MI* myocardial infarction, *PE* pulmonary embolus, *GERD* gastroesophageal reflux disease)

Using a paired t‑test we confirmed a positive change in students’ knowledge application scores from pre (M = 4.59, SD = 1.17) to post (M = 5.09, SD = 1.25), t (104) = 3.10, *p* < 0.01, with an effect size of d = 0.29. The qualitative change in how clerks conceptualize the generation of differential diagnoses was more noteworthy. Two examples of the largest transitions between pre and post student responses are shown in Fig. 1 and 2. Prior to the teaching shifts, Student A produced a list that lacked two of the three ‘can’t miss’ diagnoses in the case (pulmonary embolus and aortic dissection). It also appears that the student incorrectly extrapolated a history of trauma into the differential list from the physical finding of chest wall tenderness. The post intervention differential diagnosis list contained all of the ‘can’t miss’ diagnoses and introduced a further degree of understanding by prioritizing the list. The pre-teaching shift list described by Student B was very narrow in scope, containing differential diagnoses involving only the cardiovascular system. After the teaching shifts, the list was expanded to include multiple systems while capturing all of the ‘can’t miss’ diagnoses.

## Discussion

The competing goals of educating medical students while providing timely, quality patient care are felt acutely in the academic ED. New teaching innovations must take into account not only their relation to student education but also to the patient flow processes of the department. A programme that can show success in both areas increases its chances of acceptance and sustainability.

In our study, the overall median length of stay was 5 minutes shorter when clerks were in their teaching sessions and not caring for patients in the ED. This resonates with recent research by Delaney et al. [3], who found that after adjustments for covariates, length of stay was increased by 9.5 minutes when medical students were involved in patient care. Other research has also shown improvement in length of stay when learners were not present in the ED [4, 5]. The negative effect of students and residents on throughput is not unique to emergency medicine. Residents in orthopaedics [23], opthalmology [24] and anesthesia [25] have been shown to prolong operating room time and cost. Denton and colleagues [26] found that one medical student lengthened a half-day general internal medicine clinic by 15 %.

The clinical implications of these improvements in length of stay are difficult to gauge given the approximately 4‑hour median length of stay. It is unlikely that the 5‑minute average reduction would be seen as a meaningful difference to individual patients. However, when this 5‑minute difference is applied to all patients seen during the Monday, Tuesday and Wednesday time periods when clerks were out of the ED, this translates into an annual savings of over 140 hours per month for our two hospital study sites, whose pre-intervention wait times were in the bottom 5 % of the 74 provincial EDs measured (Dreyer J. South West Local Health Integration Network ED Lead, Personal e‑mail communication with L. Shepherd, 30 March 2014.) The 20-minute reduction in block 1 translates into length of stay being shortened by approximately 615 hours per month over this three-month period.

The financial implications of these length of stay reductions are difficult to determine within the Canadian healthcare system. We were unable to acquire reliable cost estimates at the local or provincial level to calculate costs associated with changes in length of stay. However, the cost of running the programme is easily calculated. Four hundred and eighty hours of faculty instruction made up most of the cost of this programme, with programme development being primarily uncompensated. Therefore, it is impossible to argue the case for teaching shifts based solely on a cost/benefit analysis comparing faculty costs with length of stay savings.

The educational implications of the change in ED length of stay are interesting. The block 1 results may reflect the degree of clerk inexperience early in the clerkship year, when naïve clerks require substantial time and effort to integrate into the efficient provision of patient care in any clinical rotation. This problem may be exacerbated in the ED due to patient flow pressures and the lack of a teaching team structure to help absorb the impact of naïve clinical clerks on patient care. Given these factors, some might argue that our results suggest that very junior learners are an unacceptable drag to the system and should be removed from the ED altogether. We contend that this is a slippery slope in clinical training, because a temporal effect of clerks’ impact on patient care has been reported in other settings. For instance, in surgical resident training, Hosler et al. [24].found that the operative time and cost increased with trainee participation during the first half of the academic year; these increases disappeared in the second half of the year and were attributable to the trainees’ learning curve. Our results show an improvement in length of stay in blocks 2 (non-significant) and 3 (significant) with clinical clerks working in the department, which is consistent with Hosler’s results later in the academic year. Interestingly, this progress did not continue into block 4. One possible explanation would be that clinical clerks in this block, who self-selected this late in the year scheduling, had less interest and inclination towards emergency medicine and therefore worked less efficiently. However, further study is required to understand these changes.

Furthermore, the ED offers a unique learning opportunity that medical students urgently need: an opportunity to assess and manage the range of undifferentiated patient presentations that is unequalled in most other services. With this undergraduate education perspective, we would argue that this unique learning opportunity outweighs the extra time required by clinical clerks in the ED. Rather than removing them to reduce length of stay, we would argue that dedicated teaching shifts offer a viable strategy, particularly early in the clerkship year when clerks are most naïve.

Demonstrating improved ED length of stay will please ED and hospital administrators, but ED educators will also want to understand the implications of teaching shifts in their institutions. Following the teaching shifts, the self-efficacy of clinical clerks significantly increased across each of the four factors identified, with team efficacy demonstrating the most significant improvement. We expect that this was influenced by the simulation exercises in the teaching shifts which served to supplement the students’ prior minimal exposure to active team participation. The importance of demonstrating improved self-efficacy relates to the positive effect on future performance rather than the accuracy of the prediction [27]. This is supported in the surgical literature with self-efficacy relating to laparoscopic performance in obstetrics and gynaecology residents [28] and simulator performance among surgical residents [29]. Second year medical students with higher self-efficacy were more likely to perform better on an observed structured clinical exam (OSCE) than less efficacious students [30].

Recognizing the undifferentiated nature of ED patient presentations, we were especially interested in examining practical, broad-based student performance and influences.

The assessment of knowledge application had practical origins and applications. Students and faculty both found the paper-based cases a useful exercise in providing a realistic introduction to approaching the non-resuscitative ED patient. This was reflected in the significant improvement of the scores post teaching shifts. Although Boulet’s scoring rubric has been well validated in the international medical graduate cohort applying to United States graduate training programmes [20], we acknowledge that applying this method of assessment to knowledge application is novel and requires further validation. However, we believe that this application holds promise as evidenced by the qualitative review of the exercise which illustrates clear improvement in these students’ ability to generate a differential diagnosis. There was substantive improvement in both capture of critical diagnosis and increased system inclusion in the generation of differential diagnoses. Proposed investigations and treatments also showed qualitative improvement in the paper cases.

There are several potential limitations of this investigation. It was a single-centre study where medical students spend two weeks in their emergency medicine rotation and length of stay at baseline is high. Future research is required to understand the impact of dedicated teaching shifts in a centre where rotation length is four weeks (common throughout North America) and ED length of stay is lower. As in all studies exploring ED throughput times, it is impossible to control for all of the variables that effect length of stay. By examining data from Mondays, Tuesdays and Wednesdays with the same medical students over two different weeks, we were able to control many variables. However, it is possible that other variables, such as staffing patterns and availability of ancillary and consulting services, would have been different during the weekend and other days of the week, potentially altering the results. Test/retest effect of the self-efficacy questionnaire may have falsely inflated the results. It would have been ideal to conduct a multi-level analysis of these self-efficacy results to examine teacher effect but we were limited by our small group size. Finally, our exploration of knowledge application was a proof-of-concept assessment and requires validation for future use. It was not designed to objectively assess the extent or quality of student learning during the teaching shifts, and therefore this study cannot speak to those important issues. With departmental buy-in secured by this first study’s results, our future research will examine student learning in the context of our new ED clerkship format.

## Conclusions

It is possible to balance the demands of patient flow with the needs of learners using teaching shifts. Teaching shifts can have a positive relationship to length of stay, and their use with the most naïve clerks in the first quarter of the academic year appears to be of most potential benefit. Students demonstrated improved self-efficacy and knowledge application following their teaching shifts. We conclude, therefore, that while ED educational innovations come at a cost of faculty time and instructional pay, they do not have to come at the cost of patient flow or student learning.
